# Detection of VIM-1-producing *E. coli* in German cattle

**DOI:** 10.1093/jacamr/dlaf009

**Published:** 2025-02-04

**Authors:** Alexandra Irrgang, Silke Jahn, Maria Borowiak, Annemarie Kaesbohrer, Mirjam Grobbel

**Affiliations:** German Federal Institute for Risk Assessment, Department Biological Safety, Max-Dohrn-Straße 8-10, 10589 Berlin, Germany; German Federal Institute for Risk Assessment, Department Biological Safety, Max-Dohrn-Straße 8-10, 10589 Berlin, Germany; German Federal Institute for Risk Assessment, Department Biological Safety, Max-Dohrn-Straße 8-10, 10589 Berlin, Germany; German Federal Institute for Risk Assessment, Department Biological Safety, Max-Dohrn-Straße 8-10, 10589 Berlin, Germany; German Federal Institute for Risk Assessment, Department Biological Safety, Max-Dohrn-Straße 8-10, 10589 Berlin, Germany

Carbapenems are essential therapeutics for the treatment of severe human infections with MDR bacteria. Resistance mediated by degrading enzymes (carbapenemases) can spread easily as corresponding genes are usually located on mobile genetic elements. Although carbapenems are not approved in veterinary medicine, resistance genes have been detected sporadically since 2010 with increasing frequency in samples from animals, aquaculture and food.^1^

In December 2023, we detected a VIM-1-producing *Escherichia coli* from caecal content of a fattening calf at slaughter. Although VIM-1 carbapenemase has been detected in German food production before,^2,3^ this is to our knowledge the first report of the detection of carbapenemase-producing (CP) *E. coli* in German cattle.

The isolate was detected in Germany within the EU Harmonized Monitoring programme for antimicrobial resistance (AMR)^4^ from a fattening calf at a slaughterhouse by selective ESBL isolation, whereas the selective isolation for CP *E. coli* failed (both methods described in: https://www.eurl-ar.eu/CustomerData/Files/Folders/{PI}21-protocols/721_esbl-ampc-cpeprotocol-version-caecal-v8-{PI}24042024.pdf, 2025-01-10). The strain was not able to grow on the selective commercial agar used (ChromID CarbaSmart, bioMérieux; ChromArt CRE, Biolife), whereas growth on prepared MacConkey agar supplemented with 1 mg/L cefotaxime and 0.125 mg/L meropenem was observed. Interpreted by EUCAST epidemiological cut-offs (ECOFFs) (https://mic.eucast.org; 2025-01-10) the strain showed non-WT MIC values for imipenem (1 mg/L) and meropenem (0.06 to 0.125 mg/L), but not for ertapenem (0.03 mg/L) and temocillin (8 mg/L). It further showed non-WT values for ampicillin (>32 mg/L), cefotaxime (16 mg/L), ceftazidime (8 mg/L) and sulfamethoxazole (512 mg/L). As the *fosA7* gene and an additional chromosomal point mutation (glpT_E448K) were detected by AMRFinderPlus (NCBI), resistance to fosfomycin is also expected, which is not included in the MIC panels used according to the Commission Implementing Decision (EU) 2020/1729.^4^ The isolate was sequenced with short-read (Illumina NextSeq500, DNA Prep (M) Tagmentation) and long-read (Oxford Nanopore Technology, Minion Flow Cell R10.4.1; Rapid Barcoding Kit 96 V14) sequencing techniques. Hybrid assembly (Unicycler v.0.4.8) revealed a closed genome, accessible at NCBI (PRJNA1180924), which was further processed by our in-house pipeline BakCharak v.3.1.6 (https://gitlab.com/bfr_bioinformatics/bakcharak). Analysis of the sequence data showed the presence of a 100 kb IncY plasmid and of a small 16 667 bp plasmid (CP172873), on which the *bla*_VIM-1_ was located as part of a class 1 integron. This In110 integron, harbouring *bla*_VIM-1_, *aac(6′)-Ib4* and *aadA1* (AJ969234), was detected in Enterobacterales from German fattening pig production before^2,3,5^ and is known to be involved in the spread of *bla*_VIM-1_ in different Gammaproteobacteria (Blast search; NCBI). Nevertheless, there is no hint of phylogenetic relation to formerly detected VIM-1-encoding integrons from the German food chain. The class 1 integron pEC23-AB02114 was part of a Tn*3*-family transposon, whereas in isolates from the pig production chain it was part of a Tn*21* transposon.^6^

The detected resistance genes are given in Table 1. Although only slightly reduced susceptibility to carbapenems was observed, *bla*_VIM-1_ was expressed according to high MIC values of cephalosporins and no other encoded β-lactamase genes. Additionally, transformant cells showed comparable MIC values. The VIM-1 plasmid was transferable by transformation but was not self-transferable by conjugation. The *bla*_VIM-1_-harbouring plasmid was highly similar to a 14 kb plasmid detected from *Enterobacter hormachei* (ON209138.1) and *Enterobacter cloacae* (KF998104.1), both isolated from Chinese hospitalized patients in 2022 and 2014, respectively.^7^ This plasmid from *Enterobacter* sp. harboured the *bla*_VIM-1_ gene in a disrupted class 1 integron, missing *aadA1*, *sul1* and parts of *aac(6′′)-Ib4* resistance genes. This indicates that both carbapenemase-producing Enterobacterales (CPE) plasmids detected have a common ancestor whose derivatives have been circulating around the world for more than 10 years.

**Table 1.** Strain characteristics of VIM-1-producing *E. coli* detected from a fattening calf at slaughter

Isolate ID	MLST/phylogroup	Phenotypic resistance^a^	AMR genes (acquired)	Plasmid sizes [incompatibility group]
23-AB02114	847/B1	AMP, CAZ, CTX, FEP, IPM; (MEM), SMX	*aac(6′)-Ib4*; *aadA1*; *bla*_VIM-1_; *fosA7.5*; *sul1*	100 514 bp [IncY] 16 776 bp^b^

AMP, ampicillin; CAZ, ceftazidime; CTX, cefotaxime; FEP, cefepime; IPM, imipenem; MEM, meropenem (varying around ECOFF); SMX, sulfamethoxazole.

^a^MIC test panels EUVSEC2/EUVSEC3 according to CID (EU) 2020/1729.^4^

^b^ *bla*_VIM-1_-harbouring plasmid (pEC21-AB02114 Acc. No.: CP172873).

Unfortunately, the farm was not available for trace-back investigations. Later we were informed by the responsible authorities that the respective farm unit closed for economic reasons.

Since the first CPE detection in fattening pigs, concerns have been raised that livestock could become a new reservoir for CPE. So far, CP *E. coli* have been detected only sporadically from European livestock.^8^ However, in Italy, the emergence of *bla*_OXA-181_-producing *E. coli* has raised some concern recently, in both beef cattle (veal) and pig production.^9^ CPE monitoring data reflect only the tip of the iceberg, as only *E. coli* are currently included, but the mobile genetic elements can spread between bacteria from different species. So, CPE spread within the EU food production chain might be wider than sporadic findings suggest. This underestimation is reinforced by the fact that, for instance, VIM-1-producing *E. coli* from livestock display slightly reduced phenotypic carbapenem susceptibility and are therefore not always easily detected within CPE specific monitoring.^10^ It is highly likely that the first CPEs in animals did spill over from human sources. However, by becoming a new reservoir, spill-back events from animal to human are likely to occur in the future.
